# Supplementary material for: Quantification and Distribution of Thiols in Fermented Grains of Sauce-Aroma Baijiu Production Process
Source: Foods. 2023 Jul 10;12(14):2658. doi: 10.3390/foods12142658 (PMC10378441; doi:10.3390/foods12142658)
Supplement: Supplementary file 1 [file foods-12-02658-s001.zip › foods-2426814-supplementary.pdf]

Quantification and distribution of thiols in fermented grains of sauce-aroma baijiu production process

Supplementary Information

Table S1. The detail information of the MRM method for the thiol compounds

PubChem CID	Compounds	Formula	Mass	Retention Time	Parent Ion	Daughter Ion	Cone Voltage	Collision Energy
878	methanethiol	CH_4S	48.1	1.40	158.0	110.7	23	19
6343	ethanethiol	C_2H_6S	62.1	2.39	172.0	143.0	23	19
1567	2-mercapto-1-ethanol	C_2H_6OS	78.1	3.92	187.6	173.0	21	15
7363	2-furfurylthiol	C_5H_6OS	114.2	6.18	223.7	143.8	21	19
34286	2-methyl-3-furanethiol	C_5H_6OS	114.2	8.73	224.0	110.6	23	25
78126	2-phenylethanethiol	$C_8H_{10}S$	138.2	15.39	248.5	143.5	23	20

Table S2. The concentration of five thiols in fermented grains of sauce-aroma baijiu from the first to seventh rounds

Compounds	R1 (μg/kg)	R2 (μg/kg)	R3 (μg/kg)	R4 (μg/kg)	R5 (μg/kg)	R6 (μg/kg)	R7 (μg/kg)
Methanethiol	67.64±0.89	73.38±1.56	86.30±2.81	107.05±1.14	142.41±7.57	159.57±22.48	205.37±15.55
Ethanethiol	1.24±0.05	1.22±0.03	1.30±0.02	1.39±0.02	1.59±0.18	1.52±0.04	1.76±0.07
2-Mercapto-1-ethanol	1.85±0.36	1.12±0.06	1.54±0.26	1.20±0.16	1.76±0.29	1.78±0.13	1.84±0.25
2-Furfurylthiol	0.51±0.03	0.62±0.05	0.83±0.05	1.34±0.09	2.16±0.31	2.35±0.28	3.03±0.34
2-Methyl-3-furanethiol	1.92±0.29	1.70±0.24	4.23±0.62	4.57±0.61	8.43±1.41	11.14±1.53	12.74±1.73

Table S3. The concentration of five thiols in fermented grains during the whole fifth round

Compounds	Initial (μg/kg)	Heaped (μg/kg)	Fermented (μg/kg)	Distilled (μg/kg)
Methanethiol	65.48±0.18	65.00±0.17	103.78±34.07	143.19±54.29

Ethanethiol	1.15±0.01	1.15±0.00	1.17±0.02	1.21±0.03
2-Mercapto-1-ethanol	1.39±0.28	1.53±0.29	1.13±0.43	1.10±0.25
2-Furfurylthiol	0.58±0.02	0.59±0.02	1.40±0.78	1.97±0.89
2-Methyl-3-furanethiol	1.17±0.22	0.95±0.09	4.07±2.22	6.35±3.26
